# Organizational Breast Cancer Data Mart: A Solution for Assessing Outcomes of Imaging and Treatment

**DOI:** 10.1200/CCI.23.00193

**Published:** 2024-04-15

**Authors:** Margarita L. Zuley, Jonathan Silverstein, Durwin Logue, Richard S. Morgan, Rohit Bhargava, Priscilla F. McAuliffe, Adam M. Brufsky, Andriy I. Bandos, Robert M. Nishikawa

**Affiliations:** ^1^ University of Pittsburgh, School of Medicine & University of Pittsburgh Medical Center, Department of Radiology, Division of Breast Imaging, Pittsburgh, PA; ^2^ University of Pittsburgh, School of Medicine, Department of Biomedical Informatics, Pittsburgh, PA; ^3^ University of Pittsburgh, School of Medicine, Department of Radiology, Imaging Research, Pittsburgh, PA; ^4^ University of Pittsburgh, School of Medicine, Department of Pathology and Pathology at Magee-Womens Hospital of the University of Pittsburgh Medical Center, Pittsburgh, PA; ^5^ University of Pittsburgh, School of Medicine, Department of Surgery and Breast Surgical Oncology at Magee-Womens Hospital of the University of Pittsburgh Medical Center, Pittsburgh, PA; ^6^ University of Pittsburgh, School of Medicine & University of Pittsburgh Medical Center, Division of Hematology/Oncology, Pittsburgh, PA; ^7^ University of Pittsburgh, Graduate School of Public Health, Biostatistics, Pittsburgh, PA

## Abstract

**PURPOSE:**

In the United States, a comprehensive national breast cancer registry (CR) does not exist. Thus, care and coverage decisions are based on data from population subsets, other countries, or models. We report a prototype real-world research data mart to assess mortality, morbidity, and costs for breast cancer diagnosis and treatment.

**METHODS:**

With institutional review board approval and Health Insurance Portability and Accountability Act (HIPPA) compliance, a multidisciplinary clinical and research data warehouse (RDW) expert group curated demographic, risk, imaging, pathology, treatment, and outcome data from the electronic health records (EHR), radiology (RIS), and CR for patients having breast imaging and/or a diagnosis of breast cancer in our institution from January 1, 2004, to December 31, 2020. Domains were defined by prebuilt views to extract data denormalized according to requirements from the existing RDW using an export, transform, load pattern. Data dictionaries were included. Structured query language was used for data cleaning.

**RESULTS:**

Five-hundred eighty-nine elements (EHR 311, RIS 211, and CR 67) were mapped to 27 domains; all, except one containing CR elements, had cancer and noncancer cohort views, resulting in a total of 53 views (average 12 elements/view; range, 4-67). EHR and RIS queries returned 497,218 patients with 2,967,364 imaging examinations and associated visit details. Cancer biology, treatment, and outcome details for 15,619 breast cancer cases were imported from the CR of our primary breast care facility for this prototype mart.

**CONCLUSION:**

Institutional real-world data marts enable comprehensive understanding of care outcomes within an organization. As clinical data sources become increasingly structured, such marts may be an important source for future interinstitution analysis and potentially an opportunity to create robust real-world results that could be used to support evidence-based national policy and care decisions for breast cancer.

## Teaser

This institutional data mart is a template for collecting real-world breast cancer outcomes in the United States.

## INTRODUCTION

Breast cancer is the most common cancer diagnosed in women and the leading cause of cancer death in women worldwide,^1^ with estimated global macroeconomic cost of $2 trillion international dollars for 2020-2050, using 2017 prices.^2^ In the United States, breast cancer treatment accounts for 14% of all cancer costs with 2020 annual expenditure of $29.8 billion. The highest care cost occurs in the final year of life, estimated to be $76,100 per patient.^3^ Despite the magnitude of expenditures on diagnosis and treatment of this disease, an estimated 43,000 women will die in 2023 in the United States from it.^4^ Thus, identifying real-world optimal diagnosis and care strategies is critical to reduce individual and societal impacts. Such determinations are difficult because of lack of real-world data comparing different approaches for detection, diagnosis, and treatment. For a comprehensive analysis of breast cancer care in the United States, large data sets are needed, and, as outcomes are dependent on regional variances that include delivery of care and population differences, data sets from multiple institutions and regions are required to understand the status nationally.

**CONTEXT**

**Key Objective**
In the United States, what resource contains comprehensive real-world data on breast cancer? Currently no single resource contains this information. Institutions, however, do have all the data needed for their patients. We report a novel breast care research data mart that may be a template for other organizations to evaluate any question related to breast care.
**Knowledge Generated**
A research breast data mart containing over 500 discrete data elements and free-text reports having relevant information was created from multiple clinical source structures for approximately 500,000 patients. Cancer biology, treatment, and outcome details for approximately 15,000 breast cancer cases were included.
**Relevance *(J.L. Warner)***
Disease-specific data marts have the potential to lower the barrier for translational research efforts by researchers and clinicians.*
  *Relevance section written by *JCO Clinical Cancer Informatics* Editor-in-Chief Jeremy L. Warner, MD, MS, FAMIA, FASCO.

In several countries, population-based national registries collect detailed screening, treatment, and outcome data. This enables direct understanding of benefits and costs of screening and treatments in those populations. However, in the United States, screening is voluntary, and no such comprehensive registry exists. Federal law requires states maintain a cancer registry (CR). The Centers for Disease Control (CDC) oversees the National Program of Cancer Registries (NPCR), which encompasses approximately 97% of the population. The National Cancer Institute SEER program includes approximately 48% of the population. Both registries collect detailed information regarding stage at diagnosis, treatments, and outcomes, as does the American Surgical Society's Commission on Cancer (CoC) registry. None collect imaging information. The American College of Radiology's registry, the National Mammography Database, collects detailed imaging history but only has cursory data on stage at diagnosis and no outcome data.

The Breast Cancer Surveillance Consortium (BCSC) includes six active SEER registries collecting screening information and SEER data so is an important comprehensive database. The US Preventative Services Task Force guidelines, updated in 2009^5^ and 2016,^6^ have been based in part on BCSC data with estimations for population-level outcomes using NCI's Cancer Information and Modeling Network (CISNET). Critics have raised concern that because BCSC registries are in regions with relatively poor care delivery, the data do not reliably predict outcomes of screening, but instead reflects a fractured health care delivery system. Merging of SEER, NPCR, NMD, and BCSC is possible but challenging because of significant privacy concerns regarding data sharing, costs in creating and maintaining a new database, and other factors.

Institutions have invested significantly in electronic storage of medical information in the past several decades. Most have multiple products intended to collect portions of information such as radiology (RIS), pathology (PD), and medical oncology (MD) information systems, electronic health records (EHRs), and CRs. Although each serves a particular purpose for patient care, historically these have been siloed. Recently, institutions have begun to realize the powerful clinical care, quality improvement, and clinical research benefits that internal data warehouses can provide. Large-scale initiatives such as from the Office of the National Coordinator for Health Information Technology (ONC) also are moving health care vendors and organizations toward ever increasing standardization to facilitate information exchange for optimal patient care. This initiative and others will ultimately facilitate easier sharing across institutional data marts for other purposes such as research.

Herein, we present the process involved in creation of a research institutional breast data mart. The mart's purpose is to study innumerable questions related to breast health and care delivery and to be a template for other organizations.

## METHODS

### Mart Construction

Institutional review board approval was obtained to create and study a Health Insurance Portability and Accountability Act (HIPPA)-compliant research breast data mart within our preexisting research data warehouse (RDW), Neptune. The RDW was the resource for cohort identification and data extraction. It contains atomic layer data stored close to source structures, containing only transformations for deidentification requirements, and extracted from our EHR systems, health insurance claims, and research data. Data are extracted monthly from the source structures for transformation and loading into the RDW, the construction of which has been previously published.^7^ This design allows the same high level of granularity as the source systems, thereby creating a gold standard that would be lost if the data were manipulated or rolled up in any way. In cases where domain data are pulled from multiple source systems, the domain data are stored in a table specific to that domain and source system, thereby permitting researchers to select the data from the preferred source system. As new elements are adopted by source structures and added to RDW, the mart will be updated as well. Thus, the mart represents an evolving clinical database that will stay current with clinical systems.

A working group of radiology, pathology, surgical, medical, and radiation oncology physicians, the chief informatics research officer, computer scientists, and the cancer registrar itemized a comprehensive list of data elements and their source information system(s) related to patient demographics, imaging, diagnosis, treatments, costs, and outcomes.

At outset, the RDW did not contain RIS (ImageCast, General Electric, Waukesha, WI) and CR (METRIQ, Eleckta Inc, Stockholm, Sweden) content. Appropriate leaders were interviewed to understand their databases and data transfer agreements established. For the CR, all persons with an International Classification of Diseases (ICD)-9 or ICD-10 code indicating a breast cancer diagnosis (IDC-9: 174.0-174.9,198.81, 233 and ICD-10: C50.011-50.919, C79.81, D05.90, D05.91, D05.92) who underwent initial therapy at our primary breast care facility (Magee Womens Hospital of University of Pittsburgh Medical Center) were included for initial trial mart construction. North American Association of Central Cancer Registries (NAACCR)–required elements and several site-specific elements were included (Appendix Table A1). All RIS elements in the source structure were incorporated. Data dictionaries were collected for both the CR and RIS.

PD (CoPath Plus, Sunquest, Tucson, AZ) and MD (Aria, Varian Inc, Palo Alto, CA) information were in the RDW before this effort; however, the data exist as free text. For example, synoptic surgical pathology reports that contained detailed information on every cancer had not been discretely identified nor evaluated for potential mining previously. To improve the RDW search for these elements, interviews with the pathology informatics and physician leaders clarified the report constructs. Then, an RDW analysis identified these reports, determined what structured data were available, and what data existed as free text.

### Data Collection

In an iterative process, every possible source location for each element was identified and evaluated. When possible, the primary element source was the one most frequently completed, robust, and extractable. As examples, EHR and RIS included family history. We compared element fill rates and specific relationship information. The EHR was selected because of better granularity (eg, EHR had paternal uncle, whereas RIS only contained uncle). In another example, cancer immunohistochemistry elements (ie, estrogen receptor, progesterone receptor and Her-2-neu) were in the PD and CR. The CR was selected as primary as it contained discrete site-specific data elements, whereas the PD contained free text only in the synoptic surgical pathology reports. To mine free text, an algorithm such as natural language processing (NLP) is necessary. All available sources for every element were included in the mart regardless of which was denoted as primary, then elements were sorted and labeled into related groups.

### Mart Organization

The RDW was queried for all patients with at least one breast imaging examination (on the basis of RIS examination codes) from January 1, 2004, through December 31, 2020. Each patient was assigned a unique research identifier. Oracle (Oracle, Inc, Austin, TX) was used for the mart construction. Data domains were defined by prebuilt views of grouped elements to extract data denormalized according to RDW requirements. The views were used to add data using an export, transform, load pattern from the RDW to the mart.

**FIG 1.**
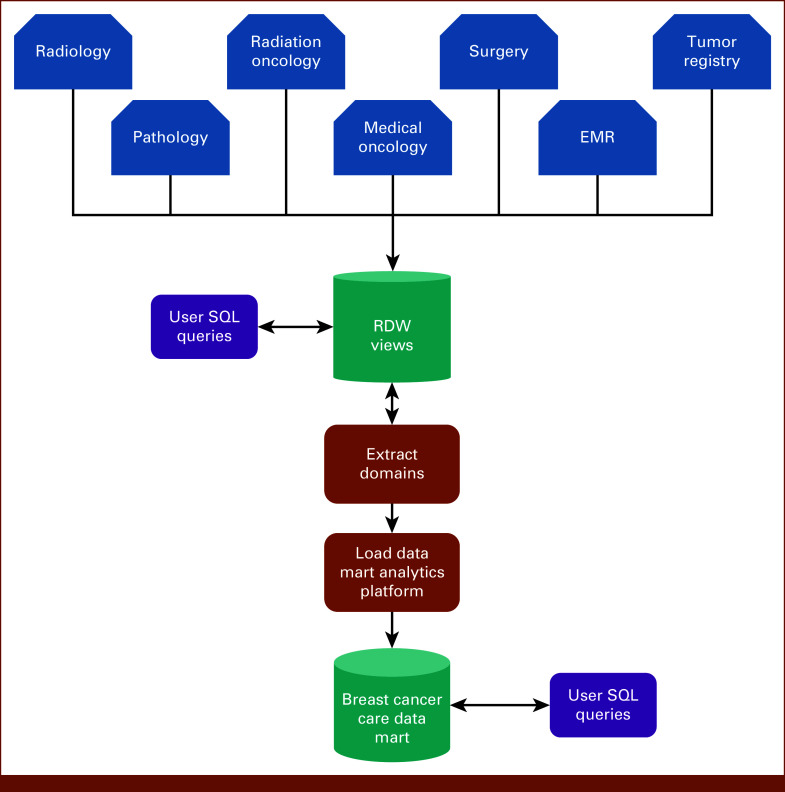
Data flow diagram. Neptune is the name of our research data warehouse. EMR, electronic medical record; RDW, research data warehouse; SQL, structured query language.

The mart was structured into two cohorts so that domains (except the CR) contained two views. Cohort” view included everyone with an ICD-9 or ICD-10 diagnosis code for breast cancer; control view included everyone with a RIS examination code specific for a breast imaging examination and without these ICD-9/-10 codes. Each Oracle view, imported element, source structure name, and comments from the RDW building team (if needed) were cataloged in the mart for researcher reference. Figure 1 depicts the data flow. Appendix Table A1 lists all mart data elements and views.

### Initial Mart Quality Analysis

Structured query language searches sorted the number of examinations by year and compared results against the historic number of examinations performed in the organization to filter and consolidate duplicate records. Valid mammogram dates (ie, January 1, 2004, to January 1, 2021) linked an examination to a CR entry to determine screening interval. Examinations with invalid or unknown dates were removed. Remaining examinations were then sorted by patient research ID. Screening examinations were identified by screening indication examination codes or examinations more than 260 days from the most recent previous examination date. Screen interval of a minimum of 260 days was selected to avoid inclusion of examinations that may have been performed for 6-month short-interval follow-up from the last screen. This generated an imaging history for each patient. Nonduplicates were concatenated, separated with a semicolon then joined to the CR view using patient ID and examination date as links with the CR element date of first contact. Results were pivoted to arrange all examinations for each patient in chronological order, with the associated columns for each examination.

## RESULTS

### Data Collection, Data Elements, and Domain Mapping

RDW query identified 497,218 unique patients with at least one breast imaging examination and a total of 2,967,364 breast imaging examinations. Electronic medical record query identified 39,860 patients with a diagnosis of breast cancer, based on ICD-9/-10 codes (Table 1). CR query revealed 15,619 patients who received their initial breast cancer treatment at our primary breast care facility.

**TABLE 1.** Demographics of Data Mart Population

Demographic	Screening Examinations (n = 2,068,144)	Diagnostic Examinations (n = 296,023)	Patients With a Breast Cancer Diagnosis Code (n = 39,860)	Total Population (N = 497,218)
Race, No. (%)				
White	1,856,144 (90)	259,973 (88)	36,412 (91)	440,001 (88)
African American	154,814 (7)	25,902 (9)	2,720 (7)	39,978 (8)
Asian	18,542 (1)	3,508 (1)	281 (1)	5,732 (1)
Other	3,969 (<1)	712 (<1)	67 (<1)	1,266 (<1)
Unknown	25,675 (1)	2,928 (1)	380 (1)	10,241 (2)
Ethnicity, No. (%)				
Hispanic	9,189 (<1)	2,079 (1)	132 (<1)	3,381 (1)
Non-Hispanic	1,952,146 (94)	276,362 (93)	28,180 (71)	467,304 (94)
Unknown	106,809 (5)	17,582 (6)	1,548 (4)	26,533 (5)

The working group defined 331 unique data elements and mapped them to 27 data domains. Because each domain, except the CR, included control and cohort views, the mart contains 589 total elements mapped to 53 Oracle views with an average of 12 elements per view (range, 4-67; Appendix Table A1). Source system contributions included EHR: 17 domains, 34 views, and 311 elements; RIS: nine domains, 18 views, and 211 elements; and CR: one domain, one view, and 67 elements.

### Data Curation

Data were curated by consolidating records for a given element that appeared in multiple sources and/or data were missing. For example, the RIS and EHR had menopausal status. The RIS element was the most complete, thus used when available, but when empty, we implemented a classification strategy to establish menopausal status at the time of each imaging examination. The order of analysis was used as a hierarchical decision tool with the first considered most robust and the 6^th^ the least robust. When elements conflicted, related fields were searched to attempt to establish truth. Occasionally, menopausal status was recorded as postmenopausal then later as premenopausal. In this situation, the related RIS element last menstrual period was reviewed and if blank, then EHR drug lists were used to determine if perhaps the patient was on birth control (implying premenopausal status) or prescribed drugs used for cancer treatment in some postmenopausal women (eg, aromatase inhibitors). Thus, all records except 39,123/2,967,364 (1.3%) were assigned premenopausal or postmenopausal with the residual labeled as perimenopausal. Table 2 lists steps and results.

**TABLE 2.** Menopausal Status

Category Hierarchy	Records	Premenopausal	Postmenopausal	Source View	Source Field
Declared age of menopause	1,258,195	71,327	1,186,868	Hormonal mens^a^	Age menopause
Date of last period	241,985	136,131	105,854	Hormonal mens^a^	Last menstrual date
Listed status in medical record	302,590	161,464	141,126	Hormonal mens^a^	Menstrual status
Medication indicates status^b^	3,448	2,009	1,439	Hormonal mens^a^	Entry name with age of use
Age at examination—younger than 46 or older than 54 years	49,066	25,349	23,717	Patient demographics	Date of birth
Unknown treated as perimenopausal	39,123	NA	NA	NA	NA

Abbreviation: NA, not applicable.

a Hormonal treatments and menstrual data.

b Premenopausal if tamoxifen or raloxifene in medication list at the time of examination. Postmenopausal if aromatase inhibitors in medication list at the time of examination.

## DISCUSSION

This effort builds on earlier proof-of-concept work in which we matched RIS and CR records of 1,316 patients with breast cancer.^8^ Although CDC, SEER, BCSC, CoC, and insurance databases such as Optimum each maintain some elements of the patient record, none contain the detail and breadth described herein. By linking patient information from the EHR, CR, RIS, PD, and MD, we created a breast cancer data mart that exists within our organizational RDW. This robust mart has advantages over other existing marts because information on breast imaging procedures and diagnoses are coupled with patient clinical data, treatments, and outcomes.

A strength of real-world data is that they include the source systems with every detail of the medical record. Therefore, evolving treatments, imaging modalities, etc. can be captured at the patient and encounter level to observe outcomes effects and answer important clinical questions. For example, racial disparities in outcomes can be examined at a more granular level and risk assessment can be modeled using a variety of data. In radiology, questions regarding the frequency and modality of screening and the ideal age to start and stop screening can be examined in terms of patient outcomes and economic cost-benefits. In pathology, rare breast cancer subtypes can be extracted for study of the clinical-pathologic features and outcomes to better define these entities. One could also extract granular pathology information including semiquantitative receptor data and automatically compute multivariable models such as Magee Equations to assess clinical outcomes. In breast surgical oncology, the effect of aggressive surgical intervention versus de-escalation could be carefully studied. For example, identification of populations for which surgery or axillary staging could be safely omitted might be possible, including in those receiving neoadjuvant therapies. These can be a cost-effective way of providing outstanding patient care, which can be of interest to many integrated health systems.

Although significant standard structure exists in clinical data sources on the basis of existing standards (eg, HL-7, NAACCR) and federal initiatives (eg, ONC), interpretation of standards, implementation of optional components, robust methodology to establish truth when conflicting source data are present, and a vast amount of information existing as free text all pose future challenges to navigate. For example, because our institution is within an NPCR state, the CR collects all NAACCR-required elements but not all additional SEER-required elements. For example, SEER but not NPCR requires the NAACCR element PR summary. The information is in our pathology data source as free text, thus can be identified using word search or NLP and extracted into the mart.

There are three published reports of breast cancer marts. Nelson and Weerasinghe^9^ collected data from 2008 to 2011, accumulating 250,968 mammograms for a quality improvement project. Two more recent efforts were created to facilitate research using artificial intelligence. GENERATOR supports breast cancer pathways of care at Gemelli University Hospital in Rome, Italy.^10^ This mart does not include RIS information, so it is not possible to determine the effects of imaging history or method of cancer detection on patient outcome. The Diagnosis Data Archive at Salah Azaiez University Hospital in Tunisia does contain mode of detection.^11^ The status of that effort is unclear as no detailed information, such as the number of cases, is reported yet.

Future expansion to include all images, CR from every facility, and financial and genetics information is planned. Given the amount of unstructured clinical data (eg, provider notes, procedural notes, and pathology reports) available across source systems, future work will include incorporating more unstructured data into the mart. This could be accomplished using a tool, such as MetaMap,^12^ to recognize clinical concepts in unstructured data and map these concepts to the UMLS Metathesaurus.^13^ Such an approach will allow for the codification of unstructured data, potentially expanding the scope and amount of data in the mart and aiding in the data curation. The size of our mart facilitates use of artificial intelligence, in particular deep learning, to discover new knowledge such as identifying imaging biomarkers to predict tumor response to different treatments.

We experienced several unexpected challenges. Our RIS is based on an older software program, which is harder to mine. We overcame this through a series of meetings between the RDW and RIS leaders to understand data structure details and how to best integrate them. The CR is a network of facility-level registries that migrated to the METRIQ platform at various times over the past several decades. We chose to focus, in this trial analysis, on our primary breast cancer facility as it was comprehensive of the date range and a test for incorporation of the CR more comprehensively into the warehouse. This restriction reduced available cancers for initial analysis to 39% total in the organization.

A limitation of this work is that it comes from a single health care enterprise. Our patient population does not necessarily reflect the distribution of patients across the country in terms of ethnicity, race, and social determinates of health. Nevertheless, our institution is a mixture of academic and community facilities in rural, suburban, and urban settings. It is our hope that our mart will inform other health care enterprises to develop their own mart, and as such, consensus might be achieved on elements for inclusion, thereby facilitating a collection of data marts, which, in turn, may support existing established and burgeoning national endeavors such as NCI's SEER and BOLD, so that real-world data can be studied to inform clinical decisions and national standards. This effort represents only initial internal steps. Much additional work and collaboration is needed within and across institutions and organizations to accomplish such lofty goals.

To understand real-world benefits, efficiencies and harms of breast cancer screening and treatment in the United States, patient-level linkage of demographic data, imaging history, and results with treatment, cost, and outcomes is needed. Institutions have this information disparately located in many databases. We have demonstrated the creation of a curated data set from disparately located clinical sources into a mart is possible, and we have given a detailed description that should enable any institution to replicate our data mart using their own electronic medical records. Institutional marts may play an important role eventually in understanding real-world outcomes for breast care.

## APPENDIX

**TABLE A1.** Cancer Registry Data Elements

Object	Source Table	Data Mart Column	Source Column	Comments
Clinical findings CLINICAL_FINDINGS_IE_VW, CLINICAL_FINDINGS_IE_CNTRL_VW	IMG_Clinical_Finding_IE	PATIENT_STUDY_ID	PERSON_ID	Research identifier of patient
		Accession_Number	ACCESSION_NUMBER	
		Internat_Exam_ID	INTERNAL_EXAM_ID	
		Patient_ID	Patient_ID	
		Idxrad_Exam_ID	IDXRAD_EXAM_ID	
		Clinical_Finding_DE	CLINICAL_FINDING_DE	
		Clinical_Finding_CD	CLINICAL_FINDING_CD	
		Clinical_Finding_Name	CLINICAL_FINDING_NAME	
		Exam_Completed_Date	COMPLETED_DATE	‘MM/DD/YYYY’
Diagnosis DIAGNOSIS_VW, DIAGNOSIS_CNTRL_VW	Diagnosis	PATIENT_STUDY_ID	PERSON_ID	Research identifier of patient
		Diagnosis_Code	DX_CODE	
		Diagnosis_Type	DX_CODE_REF.CODE_TYPE	Reference lookup for dx code type
		Diagnosis_Name	DX_CODE_REF.CODE_DESCRIPTION	Reference lookup for dx code name
		Diagnosis_From_Date	DX_FROM_DATE	Begin encounter level assignment of DX
				‘MM/DD/YYYY’
		Diagnosis_To_Date	DX_TO_DATE	End encounter level assignment of DX
				‘MM/DD/YYYY’
		Primary_Diagnosis_YN	PRIMARY_DX_IND	‘Y’ or ‘N’
Discharge summary DISCHARGE_SUMMARY_VW, DISCHARGE_SUMMARY_CNTRL_VW	Enc_Notes, Enc_Notes_Text	Patient_Study_ID	PERSON_ID	Research identifier of patient
			VISIT.HOSP_ADM_DATE	
		Admission_Date		‘MM/DD/YYYY HH24:MI:SS’
			VISIT.START_DATE	
		Discharge_Date	VISIT.HOSP_DISCHG_DATE	‘MM/DD/YYYY HH24:MI:SS’
			VISIT.END_DATE	
		Note_Contact_Date	CONTACT_DATE	‘MM/DD/YYYY’
		Note_CSN_ID	NOTE_CSN_ID	Unique note identifier
		Line_Num	LINE_NUM	
		Note_Text	NOTE_TEXT	
Encounter ENCOUNTER_VW, ENCOUNTER_CNTRL_VW	Visit	Patient_Study_ID	PERONS_ID	Research identifier of patient
		Visit_Start_Date	START_DATE	‘MM/DD/YYYY HH:MI:SS’
		Vist_End_Date	END_DATE	‘MM/DD/YYYY HH:MI:SS’
		Encounter_Type	CODE_REF.CODE_TITLE	Reference lookup for ‘ENC_TYPE’
		Facility	CODE_REF.CODE_TITLE	Reference lookup for ‘LOCATION’
		Appointment_Status	CODE_REF.CODE_TITLE	Reference lookup for ‘APPT_STATUS’
		Admit_Source	CODE_REF.CODE_TITLE	Reference lookup for ‘ADMIT_TYPE’
		Hospital_Service	CODE_REF.CODE_TITLE	Reference lookup for ‘HOSPITAL_SERVICE’
		Patient_Type	CODE_REF.CODE_TITLE	Reference lookup for ‘PATIENT_TYPE’
		Patient_Class	CODE_REF.CODE_TITLE	Reference lookup for ‘PATIENT_CLASS’
		Chief_Complaint	CHIEF_COMPLAINT	Free text data capture
		Chief_Complaint_Onset_Date	CHIEF_COMPLAINT_ONSET_DATE	‘MM/DD/YYYY’
		Discharge_Disposition	CODE_REF.CODE_TITLE	Reference lookup for ‘DISCHARGE_DISP’
		Financial_Class	CODE_REF.CODE_TITLE	Reference lookup for ‘FIN_CLASS’
Enter edit findings ENTEREDIT_FINDINGS_VW, ENTEREDIT_FINDINGS_CNTRL_VW	IMG_EnterEdit_Findings	Patient_Study_ID	PERSON_ID	Research identifier of patient
		Accession_Number	ACCESSION_NUMBER	
		Org_Code	ORG_CODE	
		Internal_Exam_ID	INTERNAL_EXAM_ID	
		Patient_ID	PATIENT_ID	Imagecast Patient Identifier
		Composition_Name	COMPOSITION_NAME	
		Finding_Location	FINDING_LOCATION	
		Finding_Category	FINDING_CATEGORY	
		Finding_Rec	FINDING_REC	
		Exam_Complete_Date	COMPLETED_DATE	MM/DD/YYYY
Family history AMILY_HX_VW, FAMILY_HX_CNTRL_VW	Family_HX	Patient_Study_ID	PERSON_ID	Research identifier of patient
		Line_Num	LINE_NUM	Identify each line in history
		Contact_Date	CONTACT_DATE	‘MM/DD/YYYY’
		Medical_HX_Title	CODE_REF.CODE_TITLE	Reference lookup for ‘MEDICAL_HX’
		Relation_Title	CODE_REF.CODE_TITLE	Reference lookup for ‘RELATION’
Hormonal mens HORMONAL_MENS_VW, HORMONAL_MENS_CNTRL_VW	IMG_Hormonal_Mens	Patient_Study_ID	PERSON_ID	Research identifier of patient
		Accession_Number	ACCESSION_NUMBER	
		Internal_Exam_ID	INTERNAL_EXAM_ID	
		Patient_ID	PATIENT_ID	
		Entry_Name	ENTRY_NAME	
		Age_First_Use	AGE_FIRST_USE	
		Age_Last_Use	AGE_LAST_USE	
		Duration	DURATION	
		Current_Use_IND	CURRENT_USE_IND	
		Never_Use_IND	NEVER_USE_IND	
		Age_Menarche	AGE_MENARCHE	
		Age_First_Live_Birth	AGE_FIRST_LIVE_BIRTH	
		Age_Menopause	AGE_MENOPAUSE	
		Age_Hysterectomy	AGE_HYSTERECTOMY	
		Age_Right_Ovary_Removal	AGE_RIGHT_OVARY_REMOVAL	
		Age_Left_Ovary_Removal	AGE_LEFT_OVARY_REMOVAL	
		Parity_Count	PARITY_COUNT	
		Pregnancy_Count	PREGNANCY_COUNT	
		Last_Menstrual_Date	LAST_MENSTRUAL_DATE	‘MM/DD/YYYY’
		Cycle_Phase	CYCLE_PHASE	
		Pregnant_IND	PREGNANT_IND	
		Menstrual_Status_CD	MENSTRUAL_STATUS_CD	
		Exam_Completed_Date	COMPLETED_DATE	‘MM/DD/YYYY’
IMG procedures IMG_PROCEDURES_VW, IMG_PROCEDURES_CNTRL_VW	IMG_Procedures	Patient_Study_ID	PERSON_ID	Research identifier of patient
		Accession_Number	ACCESSION_NUMBER	
		Internal_Exam_ID	INTERNAL_EXAM_ID	
		Patient_ID	PATIENT_ID	
		Procedure_Side	PROCEDURE_SIDE	
		Procedure_Date	PROCEDURE_DATE	‘MM/DD/YYYY’
		Procedure_Outcome	PROCEDURE_OUTCOME	
		Implant_Side	IMPLANT_SIDE	
		Implant_Type	IMPLANT_TYPE	
		Exam_Completed_Date	COMPLETED_DATE	‘MM/DD/YYYY’
Laboratory results LAB_RESULT_VW, LAB_RESULT_CNTRL_VW	Lab_Result	Patient_Study_ID	PERSON_ID	Research identifier of patient
		Result_Date	RESULT_DATE	‘MM/DD/YYYY HH:MI:SS’
		Component_Name	COMPONENT_NAME	
		Result_Value	ORD_VALUE	
		Result_Unit	REFERENCE_UNIT	
		Reference_Low	REFERENCE_LOW	
		Reference_High	REFERENCE_HIGH	
		Lab_Result_Status	CODE_REF.CODE_TITLE	Reference lookup for ‘RESULT_STATUS’
		Specimen_Collected_Date	SPECIMEN_COLLECTED_DATE	‘MM/DD/YYYY HH:MI:SS’
		Specimen_Received_Date	SPECIMEN_RECEIVED_DATE	‘MM/DD/YYYY HH:MI:SS’
		Specimen_Type	CODE_REF.CODE_TITLE	Reference lookup for ‘SPECIMEN_TYPE’
		Specimen_Source	CODE_REF.CODE_TITLE	Reference lookup for ‘SPECIMEN_SOURCE’
Laboratory sensitivity LAB_SENSITIVITY_VW	Lab_Sensitivity	Patient_Study_ID	PERSON_ID	Research identifier of patient
		Contact_Date	CONTACT_DATE	‘MM/DD/YYYY HH:MI:SS’
		Result_Date	RESULT_DATE	‘MM/DD/YYYY HH:MI:SS’
		Organism	ORANISM_NAME	
		Antibiotic	CODE_REF.CODE_TITLE	Reference lookup for ‘ANTIBIOTIC’
		Suscept	CODE_REF.CODE_TITLE	Reference lookup for ‘SUSCEPT’
		Sensitivity_Value	SENSITIVITY_VALUE	
		Sensitivity_Units	SENSITIVITY_UNITS	
		Sensitivity_Status	CODE_REF.CODE_TITLE	Reference lookup for ‘RESULT_STATUS’
Medication fill MED_FILL_VW, MED_FILL_CNTRL_VW	Med_Fill	Patient_Study_ID	PERSON_ID	Research identifier of patient
		Filled_Date	FILLED_DATE	‘MM/DD/YYYY’
		Drug_Name	DRUG_NAME	
		Simple_Generic_Name	SIMPLE_GENERIC_NAME	
		Drug_Code_Sys	DRUG_CODE_SYS	
		NDC	NDC	
		Amount	AMOUNT	
		Med_Units	MED_UNIT_TXT	
		Med_Unit_Strength	MED_UNIT_STRENGTH	
		Quantity	QUANTITY	
		Days_Supply	DAYS_SUPPLY	
Medication order MED_ORDER_VW, MED_ORDER_CNTRL_VW	Med_Order	Patient_Study_ID	PERSON_ID	Research identifier of patient
		Order_Date	ORDER_DATE	‘MM/DD/YYYY HH:MI:SS’
		Medication_ID	MEDICATION_ID	
		Medication	MEDICATION_NAME	
		Simple_Generic_Name	CODE_REF.CODE_TITLE	Reference lookup for ‘SIMPLE_GENERIC’
		Pharm_Class	CODE_REF.CODE_TITLE	Reference lookup for ‘PHARM_CLASS’
		Dose	DOSE	
		Med_Units	MED_UNIT_TITLE	
		Quantity	QUANTITY	
		Refills	REFILLS	
		Start_Date	START_DATE	‘MM/DD/YYYY HH:MI:SS’
		End_Date	END_DATE	‘MM/DD/YYYY HH:MI:SS’
		Admin_Route_Title	ADMIN_ROUTE_TITLE	
		Frequency	FREQUENCY	
		Instructions	INSTRUCTIONS	
Order result ORDER_RESULT_VW, ORDER_RESULT_CNTRL_VW	Order_Result	Patient_Study_ID	PERSON_ID	Research identifier of patient
		Order_Procedure_ID	ORDER_PROC_ID	
		Result_Date	RESULT_DATE	‘MM/DD/YYYY HH:MI:SS’
		Component_Name	COMPONENT_NAME	COMPONENT_REF
		Result_Value	ORD_VALUE	
		Result_Unit	REFERENCE_UNIT	
		Reference_Low	REFERENCE_LOW	
		Reference_High	REFERENCE_HIGH	
		Result_Flag_Title	CODE_REF.CODE_TITLE	Reference lookup for ‘RESULT_FLAG’
		Result_Status	CODE_REF.CODE_TITLE	Reference lookup for ‘RESULT_STATUS’
		Specimen_Collected_Date	SPECIMEN_COLLECTED_DATE	‘MM/DD/YYYY HH:MI:SS’
		Specimen_Received_Date	SPECIMEN_RECEIVED_DATE	‘MM/DD/YYYY HH:MI:SS’
Patient data IE PATIENT_DATA_IE_VW, PATIENT_DATA_IE_CNTRL_VW	IMG_Patient_Data_IE	Patient_Study_ID	PERSON_ID	Research identifier of patient
		Order_Procedure_ID	ACCESSION_NUMBER	
		Result_Date	ORG_CODE	
		Component_Name	ORG_NAME	
		Result_Value	EXAM_CODE	
		Result_Unit	EXAM_NAME	
		Reference_Low	EXAM_MODIFIER	
		Reference_High	EXAM_STATUS	
		Result_Flag_Title	SCHEDULED_DATE	‘MM/DD/YYYY’
		Result_Status	COMPLETED_DATE	‘MM/DD/YYYY’
		Specimen_Collected_Date	FINALIZED_DATE	‘MM/DD/YYYY’
		Specimen_Received_Date	BEGIN_DATE	‘MM/DD/YYYY’
		Order_Date	ORDER_DATE	‘MM/DD/YYYY’
		Exam_Type_ID	EXAM_TYPE_ID	
		Exam_Side	EXAM_SIDE	
		Exam_Requestor	EXAM_REQUESTOR	
		Exam_Type	EXAM_TYPE	
		Self_Request_Ind	SELF_REQUEST_IND	
		Equipment_Clean_Ind	EQUIPMENT_CLEAN_IND	
		First_Mammo_Ind	FIRST_MAMMO_IND	
		Last_Screen_Month_CD	LAST_SCREEN_MONTH_CD	
		Last_CBE_CD	LAST_CBE_CD	
		Last_CBE_Date	LAST_CBE_DATE	‘MM/DD/YYYY’
		Last_Activity_Date	LAST_ACTIVITY_DATE	‘MM/DD/YYYY’
		Internal_Exam_ID	INTERNAL_EXAM_ID	
		Patient_ID	PATIENT_ID	
Patient demographics PATIENT_DEMO_VW, PATIENT_DEMO_CNTRL_VW	Patient_Demographic	Person_ID	PATIENT_STUDY_ID	Research identifier of patient ‘MM/DD/YYYY’ default to July 1^st^ of birth year
		Birth_Date	BIRTH_DATE	‘MM/DD/YYYY’
		Death_Date	DEATH_DATE	Reference lookup for ‘GENDER’
		Gender_Title Race_Title	CODE_REF.CODE_TITLE	Reference lookup for ‘RACE’
		Ethnic_Title	CODE_REF.CODE_TITLE CODE_REF.CODE_TITLE	Reference lookup for ‘ETHNIC_GROUP'
Pathology PATHOLOGY_VW, PATHOLOGY_CNTRL_VW	IMG_Pathology	Patient_Study_ID	PERSON_ID	Research identifier of patient
		Bx_ID	BX_ID	
		Pathology_Date	PATHOLOGY_DATE	MM/DD/YYYY
		Patient_ID	PATIENT_ID	
		Site_Number	SITE_NUMBER	
		Side	SIDE	
		Lesion_Class	LESION_CLASS	
		Lesion_Location	LESION_LOCATION	
		Technique	TECHNIQUE	
			Comments	COMMENTS
				
Pathology findings PATHOLOGY_FINDINGS_VW, PATHOLOGY_FINDINGS_CNTRL_VW	IMG_Pathology_Findings	Patient_Study_ID	PERSON_ID	Research identifier of patient
		Bx_ID	BX_ID	
		Pathology_Date	PATHOLOGY_DATE	MM/DD/YYYY
		Patient_ID	PATIENT_ID	
		Finding_ID	FINDING_ID	
		Pathology_CD	PATHOLOGY_CD	
		Lesion_Class	LESION_CLASS	
		Finding_Size_1	FINDING_SIZE_1	
		Finding_Size_2	FINDING_SIZE_2	
		Measurement_Type	MEASUREMENT_TYPE	
		Histrology_Grade	HISTROLOGY_GRADE	
		Nodes_Removed	NODES_REMOVED	
		Nodes_Positive	NODES_POSITIVE	
		Margin_Status	MARGIN_STATUS	
		Estrogen_Receptor	ESTROGEN_RECEPTOR	
		Progesterone_Receptor	PROGESTERONE_RECEPTOR	
		HER2NEU	HER2NEU	
		Stage_T	STAGE_T	
		Stage_N	STAGE_N	
		Stage_M	STAGE_M	
		Stage_Num	STAGE_NUM	
		Nipples_Involved	NIPPLES_INVOLVED	
Problem list PROBLEM_LIST_VW, PROBLEM_LIST_CNTRL_VW	Problem_List	Patient_Study_ID	PERSON_ID	Research Identifier of Patient
		Diagnosis_Code	DX_CODE	
		Diagnosis_Type	DX_CODE_TYPE	
		Diagnosis_Name	DX_NAME	
		Reported_Date	REPORTED_DATE	‘MM/DD/YYYY’
		Onset_Date	ONSET_DATE	‘MM/DD/YYYY’
		Resolved_Date	RESOLVED_DATE	‘MM/DD/YYYY’
		Resovled_Reason	RESOLVED_REASON	
		Status_Title	CODE_REF.CODE_TITLE	Reference lookup for ‘PROBLEM_STATUS’
Procedures PROCEDURES_VW, PROCEDURES_CNTRL_VW	Procedures	PATIENT_STUDY_ID	PERSON_ID	Research identifier of patient Order listed sequence finding first not null value:
		Procedure_Date	RESULT_DATE, PROC_DATE, ORDER_DATE	‘MM/DD/YYYY’
		Procedure_Code	PROC_CODE	
		Procedure_Type	PROC_CODE_REF.CODE_TYPE	
		Procedure_Name	PROC_CODE_REF.CODE_DESCRIPTION	
		Procedure_Location	PROCEDURE_LOCATION	
		Order_Date	ORDER_DATE	‘MM/DD/YYYY’
Procedure notes PROCEDURES_NOTES_VW, PROCEDURES_NOTES_CNTRL_VW	Proc_Notes, Proc_Notes_Text	PATIENT_STUDY_ID	PERSON_ID	Research identifier of patient
		Order_Date	ORDER_DATE	‘MM/DD/YYYY’
		Result_Time	RESULT_DATE	‘MM/DD/YYYY HH:MI:SS’
		Procedure_Code	PROC_CODE	
		Procedure_Name	CODE_DESCRIPTION	PROC_CODE_REF
		Note_CSN_ID	NOTE_CSN_ID	
		Line_Num	LINE_NUM	
		Note_Text	NOTE_TEXT	
Recommendations RECOMMENDATIONS_VW, RECOMMENDATIONS_CNTRL_VW	IMG_Recommendation_IE	PATIENT_STUDY_ID	PERSON_ID	Research identifier of patient
		Accession_Number	ACCESSION_NUMBER	
		Internal_Exam_id	INTERNAL_EXAM_ID	
		Patient_ID	PATIENT_ID	
		Recommend_ID	RECOMMEND_ID	
		Recommend_CD	RECOMMEND_CD	
				
		Recommend_Status	RECOMMEND_STATUS	
		Recommend_Side	RECOMMEND_SIDE	
		Due_Date	DUE_DATE	‘MM/DD/YYYY’
		Exam_Completed_Date	COMPLETED_DATE	‘MM/DD/YYYY’
Risk factors RISK_FACTORS_VW, RISK_FACTORS_CNTRL_VW	IMG_Risk_Factors	Patient_Study_ID	PERSON_ID	Research identifier of patient
		Accession_Number	ACCESSION_NUMBER	
		Internal_Exam_id	INTERNAL_EXAM_ID	
		Patient_ID	PATIENT_ID	
		Risk_Factor_CD	RISK_FACTOR_CD	
		Risk_Factor_Name	RISK_FACTOR_NAME	
		Risk_Sequence	RISK_SEQUENCE	
		Relationship_CD	RELATIONSHIP_CD	
		Problem_CD	PROBLEM_CD	
		Person_Age	PERSON_AGE	
		Comments	COMMENTS	
		Exam_Completed_Date	COMPLETED_DATE	‘MM/DD/YYYY’
Social history alcohol SOCIAL_HX_ALC_VW, SOCIAL_HX_ALC_CNTRL_VW	Social_HX_Alc	Patient_Study_ID	PERSON_ID	Research identifier of patient
		Contact_Date	CONTACT_DATE	‘MM/DD/YYYY’
		Drinks_Per_Week	DRINKS_PER_WEEK	
		Drink_Type_Title	CODE_REF.CODE_TITLE	Reference lookup for ‘DRINK_TYPE’
Social history tobacco SOCIAL_HX_TOB_VW, SOCIAL_HX_TOB_CNTRL_VW	Social_HX_Tob	Patient_Study_ID	PERSON_ID	Research identifier of patient
		Contact_Date	CONTACT_DATE	‘MM/DD/YYYY’
		Smoking_Use_Title	CODE_REF.CODE_TITLE	Reference lookup for ‘SMOKE_TOB_USE’
		Tobacco_Use_Per_Day	TOBACCO_PAK_PER_DAY	
		Tobacco_Use_Years	TOBACCO_USED_YEARS	
				Translate ‘Y’ to ‘Yes’
		Cigarettes_Indicator	CIGARETTES_IND	Translate ‘N’ to ‘No’
				Otherwise NULL
				Translate ‘Y’ to ‘Yes’
		Pipes_Indicator	PIPES_IND	Translate ‘N’ to ‘No’
				Otherwise NULL
				Translate ‘Y’ to ‘Yes’
		Cigars_Indicator	CIGARS_IND	Translate ‘N’ to ‘No’
				Otherwise NULL
				Translate ‘Y’ to ‘Yes’
		Snuff_Indicator	SNUFF_IND	Translate ‘N’ to ‘No’
				Otherwise NULL
				Translate ‘Y’ to ‘Yes’
		Chew_Indicator	CHEW_IND	Translate ‘N’ to ‘No’
				Otherwise NULL
		Smokeless_Tob_Use_Title	CODE_REF.CODE_TITLE	Reference lookup for ‘SMOKELESS_TOB_USE’
Surgical pathology SURGICAL_PATH_NOTES_VW, Notes SURGICAL_PATH_NOTES_CNTRL_VW	Proc_Notes, Proc_Notes_Text	PATIENT_STUDY_ID	PERSON_ID	Research identifier of patient
		Order_Date	ORDER_DATE	‘MM/DD/YYYY’
		Result_Time	RESULT_DATE	‘MM/DD/YYYY HH:MI:SS’
		Procedure_Code	PROC_CODE	
		Procedure_Name	CODE_DESCRIPTION	PROC_CODE_REF
		Note_CSN_ID	NOTE_CSN_ID	
		Line_Num	LINE_NUM	
		Note_Text	NOTE_TEXT	
Vitals VITALS_VW, VITALS_CNTRL_VW	Vitals	Patient_Study_ID	PERSON_ID	Research identifier of patient
		Visit_ID	VISIT_ID	
		Weight	WEIGHT	
		Weight_Unit	WEIGHT_UNIT	
		Height	HEIGHT	
		Height_In	HEIGHT_IN	
		Height_Unit	HEIGHT_UNIT	
		Date_Taken	CONTACT_DATE	
		BMI	BMI	
Patient cancer registry PATIENT_CANCER_REGISTRY_VW	BCC_Cancer_Registry	Patient_Study_ID	PERSON_ID	Research identifier of patient
		HOSPITAL_ID	HOSPITAL_ID	
		SEQUENCE_NUMBER	SEQUENCE_NUMBER	
		CLASS_OF_CASE	CLASS_OF_CASE	
		CASE_STATUS	CASE_STATUS	
		DATE_OF_BIRTH	DATE_OF_BIRTH	Default to July 1^st^ of birth year
		SEX	SEX	
		SEX_DESC	SEX_DESC	
		RACE_1	RACE_1	
		RACE_1_DESC	RACE_1_DESC	
		SPANISH_HISPANIC_ORIGIN	SPANISH_HISPANIC_ORIGIN	
		SPANISH_HISP_ORIG_DESC	SPANISH_HISP_ORIG_DESC	
		PRIMARY_PAYOR_DX_DESC	PRIMARY_PAYOR_DX_DESC	
		POSTAL_CODE_AT_DX	POSTAL_CODE_AT_DX	
		COUNTY_AT_DX	COUNTY_AT_DX	
		PRIMARY_SITE	PRIMARY_SITE	
		PRIMARY_SITE_DESC	PRIMARY_SITE_DESC	
		LATERALITY_DESC	LATERALITY_DESC	
		HISTO_BEHAVE_ICDO3	HISTO_BEHAVE_ICDO3	
		HISTO_BEHAVE_ICDO3_DESC	HISTO_BEHAVE_ICDO3_DESC	
		HISTO_BEHAVE_ICDO3_DESC	HISTO_BEHAVE_ICDO3_DESC	
		CORRECTED_BEST_STAGE	CORRECTED_BEST_STAGE	
		DATE_1ST_CONTACT	DATE_1ST_CONTACT	
		COURSE_DATE_1ST_DX	COURSE_DATE_1ST_DX	
		COURSE_1ST_DXSTG_PROCFAC_DESC	COURSE_1ST_DXSTG_PROCFAC_DESC	
		COURSE_1ST_DXSTG_PROCSUM_DESC	COURSE_1ST_DXSTG_PROCSUM_DESC	
		CS_SITE_SPEC_FACTOR_1	CS_SITE_SPEC_FACTOR_1	
		CS_SITE_SPEC_FACTOR_2	CS_SITE_SPEC_FACTOR_2	
		CS_SITE_SPEC_FACTOR_15	CS_SITE_SPEC_FACTOR_15	
		AJCC_CLINICAL_T_DESC	AJCC_CLINICAL_T_DESC	
		AJCC_CLINICAL_N_DESC	AJCC_CLINICAL_N_DESC	
		AJCC_CLINICAL_M_DESC	AJCC_CLINICAL_M_DESC	
		REASON_NO_SURGERY_DESC	REASON_NO_SURGERY_DESC	
		MOST_DEFINITIVE_SURG_DATE	MOST_DEFINITIVE_SURG_DATE	
		COURSE_1ST_SURG_PRIMSITE_DESC	COURSE_1ST_SURG_PRIMSITE_DESC	
		COURSE_1ST_SURG_PRIMSUMM_DESC	COURSE_1ST_SURG_PRIMSUMM_DESC	
		COURSE_1ST_SCOPE_LN_SURG_DESC	COURSE_1ST_SCOPE_LN_SURG_DESC	
		REGIONAL_NODES_EXAM	REGIONAL_NODES_EXAM	
		REGIONAL_NODES_POSITIVE	REGIONAL_NODES_POSITIVE	
		COURSE_1ST_SURG_MARG_DESC	COURSE_1ST_SURG_MARG_DESC	
		REASON_NO_CHEMO_DESC	REASON_NO_CHEMO_DESC	
		COURSE_1ST_CHEMO_DATE	COURSE_1ST_CHEMO_DATE	
		COURSE_1ST_CHEMO_FAC_DESC	COURSE_1ST_CHEMO_FAC_DESC	
		COURSE_1ST_CHEMO_SUMM_DESC	COURSE_1ST_CHEMO_SUMM_DESC	
		REASON_NO_HORMONE_THERAPY	REASON_NO_HORMONE_THERAPY	
		COURSE_1ST_HORMONE_RX_DATE	COURSE_1ST_HORMONE_RX_DATE	
		COURSE_1ST_HORMONE_FAC_DESC	COURSE_1ST_HORMONE_FAC_DESC	
		COURSE_1ST_HORMONE_RX_SUMM	COURSE_1ST_HORMONE_RX_SUMM	
		MEDICAL_ONCOLOGY_PHYS_NPI	MEDICAL_ONCOLOGY_PHYS_NPI	
		MEDICAL_ONC_PHYS_LASTNAME	MEDICAL_ONC_PHYS_LASTNAME	
		MEDICAL_ONC_PHYS_FIRSTNAME	MEDICAL_ONC_PHYS_FIRSTNAME	
		REASON_NO_RADIATION_DESC	REASON_NO_RADIATION_DESC	
		DATE_RT_STARTED	DATE_RT_STARTED	
		DATE_RT_ENDED	DATE_RT_ENDED	
		COURSE_1ST_RADIATION_FAC	COURSE_1ST_RADIATION_FAC	
		COURSE_1ST_RT_MODALITY_SUMM	COURSE_1ST_RT_MODALITY_SUMM	
		COURSE_1ST_RT_VOLUME_SUMM	COURSE_1ST_RT_VOLUME_SUMM	
		COURSE_1ST_RT_REG_DOSE_SUMM	COURSE_1ST_RT_REG_DOSE_SUMM	
		COURSE_1ST_RX_SUMMARY	COURSE_1ST_RX_SUMMARY	
		RADIATION_ONC_PHYS_NPI	RADIATION_ONC_PHYS_NPI	
		RADIATION_ONC_PHYS_LASTNAME	RADIATION_ONC_PHYS_LASTNAME	
		RADIATION_ONC_PHYS_FIRSTNAME	RADIATION_ONC_PHYS_FIRSTNAME	
		CHEMOTHERAPY_TEXT	CHEMOTHERAPY_TEXT	
		DATE_LAST_CONTACT	DATE_LAST_CONTACT	
		VITAL_STATUS	VITAL_STATUS	
		CANCER_STATUS_DESC	CANCER_STATUS_DESC	
		DATE_1ST_RECURRENCE	DATE_1ST_RECURRENCE	
		TYPE_1ST_RECURRENCE_DESC	TYPE_1ST_RECURRENCE_DESC	
